# Incidence of Deep Vein Thrombosis and Venous Thromboembolism following TKA in Rheumatoid Arthritis versus Osteoarthritis: A Meta-Analysis

**DOI:** 10.1371/journal.pone.0166844

**Published:** 2016-12-02

**Authors:** Do-Kyung Lee, Hyun-Jung Kim, Dae-Hee Lee

**Affiliations:** 1 Department of Orthopaedic Surgery, Samsung Medical Center, Sungkyunkwan University School of Medicine, Seoul, Korea; 2 Department of Preventive medicine, Korea University College of Medicine, Seoul, Korea; Medical University Innsbruck, AUSTRIA

## Abstract

This meta-analysis was designed to compare the incidence of deep vein thrombosis (DVT) and venous thromboembolism (VTE) following total knee arthroplasty (TKA) in patients with rheumatoid arthritis (RA) and osteoarthritis (OA). All studies directly comparing the post-TKA incidence of DVT and/or VTE in patients with RA and OA were included. For all comparisons, odds ratios and 95% confidence intervals (CI) were calculated for binary outcomes. Six studies were included in the meta-analysis. The pooled data showed that the combined rates of asymptomatic and symptomatic DVT did not differ significantly in the RA and OA groups (1065/222,714 [0.5%] vs. 35,983/6,959,157 [0.5%]; OR 0.77, 95% CI: 0.57 to 1.02; P = 0.07). The combined rates of asymptomatic and symptomatic DVT and pulmonary embolism (PE) after TKA were significantly lower in the RA than in the OA group (1831/225,406 [0.8%] vs. 63,953/7,018,721 [0.9%]; OR 0.76, 95% CI: 0.62 to 0.93; P = 0.008). Conclusiviely, the DVT rates after primary TKA were similar in RA and OA patients. In contrast, the incidence of VTE (DVT plus PE) after primary TKA was lower in RA than in OA patients, despite patients with RA being at theoretically higher risk of thrombi due to chronic inflammation.

## Introduction

The vast majority of total knee arthroplasties (TKAs) have been performed in patients with advanced knee osteoarthritis (OA).[1–3] However, patients with rheumatoid arthritis (RA) and severe involvement of the knee joint may also be candidates for TKA.[4] Venous thromboembolism (VTE), including both deep vein thrombosis (DVT) and pulmonary embolism (PE), is a potentially life-threatening complication after TKA. VTE is considered more likely to develop in patients with RA than OA, because chronic inflammatory mediators in RA are associated with prothrombotic factors and endothelial dysfunction, making these patients more susceptible to the development of atherosclerosis and thrombosis.[5,6] Nonetheless, few studies to date have directly compared the incidence of VTE after TKA in patients with RA and OA, with some comparative studies showing contradictory results [7–9]. The relative incidence of VTE after TKA in patients with RA and OA is therefore unclear.

This meta-analysis was therefore designed to compare the incidence of VTE following TKA in RA and OA patients by pooling data from previous comparative studies. It was hypothesized that the incidence of post-TKA VTE would be higher in patients with RA than in patients with OA.

## Materials and Methods

This meta-analysis was performed according to the guidelines of the preferred reporting items for systematic reviews and meta-analysis (PRISMA) statement (S1 PRISMA Checklist).

### Data and literature sources

This study was based on Cochrane Review Methods. Multiple comprehensive databases, including MEDLINE, EMBASE, Web of Science, SCOPUS, and the Cochrane Library, were searched for studies that compared the rates of DVT and VTE (a composite of DVT plus PE) following primary TKA in patients with RA and OA (S1 Search strategy). There were no restrictions on language or year of publication. Search terms used in the title, abstract, MeSH, and keywords fields included "arthroplasty" [tiab] OR "replacements" [tiab] OR "knee" [tiab], and "deep vein thrombosis" [MeSH] OR "venous thromboembolism" [MeSH] OR “pulmonary embolism”[MeSH] OR "rheumatoid arthritis" [tiab] OR "osteoarthritis" [tiab]. After the initial electronic search, relevant articles and their bibliographies were searched manually. The identified articles were individually assessed for inclusion.

### Study selection

Two reviewers (DKL and DHL) independently assessed the title and abstract of identified articles and selected the relevant studies for full review. The full text copy of the article was reviewed if the abstract did not provide sufficient data to make a decision. Studies were included in the meta-analysis if they (1) assessed the incidence of post-TKA asymptomatic and/or symptomatic DVT and/or VTE in RA and OA patients; (2) directly compared the rates of DVT and VTE in RA and OA patients; and (3) reported the actual numbers of patients in the RA and OA groups and the actual numbers, not only the percentages, of patients in each group who developed DVT and/or VTE.

### Data extraction

Two reviewers (DKL and DHL) independently recorded data from each study using a predefined data extraction form. Disagreement between the reviewers was resolved by consensus or by discussion with a third investigator (HJK) when consensus was not reached. Variables recorded included blood loss and postoperative complications associated with PE and DVT, including the numbers and percentages of RA and OA patients with post-TKA asymptomatic and/or symptomatic DVT and/or VTE after primary TKA and the sample size of each group. If these variables were not mentioned in the articles, the study authors were contacted to retrieve further information.

### Assessment of methodological quality

Two reviewers (DKL and DHL) independently assessed the methodological qualities of each study using the Newcastle-Ottawa Scale, as recommended by the Cochrane Non-Randomized Studies Methods Working Group. The current meta-analysis used the adjusted Newcastle-Ottawa Scale star system, which had three criteria: the selection of study groups, the comparability of these groups and the ascertainment of either the exposure or outcome of interest for case-control and cohort studies. High quality studies were those having scores ≥5 points. Disagreements in scores were resolved by discussion and consensus between the two reviewers. Publication bias was not assessable in these trials. Tests for funnel plot asymmetry are typically conducted only when at least 10 studies are included because that number of studies is required to differentiate an asymmetry identified from chance. As our analysis included only 7 studies, tests for asymmetry were not conducted.

### Data synthesis and analysis

The main outcomes of this meta-analysis were the rates after primary TKA of asymptomatic and/or symptomatic DVT and/or VTE (DVT + PE) in patients with RA and OA. For all comparisons, odds ratios (ORs) and 95% confidence intervals (CI) were calculated for binary outcomes. These values were analyzed with a random effects model. The weights were based on a combination of the sampling error (variance of DVT and/or VTE rates within each study) and the random-effect variance (variance of DVT and/or VTE rates between all studies). Heterogeneity was determined by estimating the proportion of between-study inconsistencies due to actual differences between studies, rather than differences due to random error or chance using the I^2^ statistic, with values of 25%, 50%, and 75% considered low, moderate, and high heterogeneity, respectively. All statistical analyses were performed with RevMan version 5.2 static software. Subgroup analyses included patients with and without symptoms of DVT and/or VTE. The results of the current meta-analysis could be biased by the demographic characteristics (age and gender) of the patients in the included studies. A meta-regression analysis was therefore performed to evaluate the possible confounding effects of differences in age and gender of RA and OA patients on the rates of DVT and VTE after TKA.

## Results

### Identification of Studies

**Fig 1:**
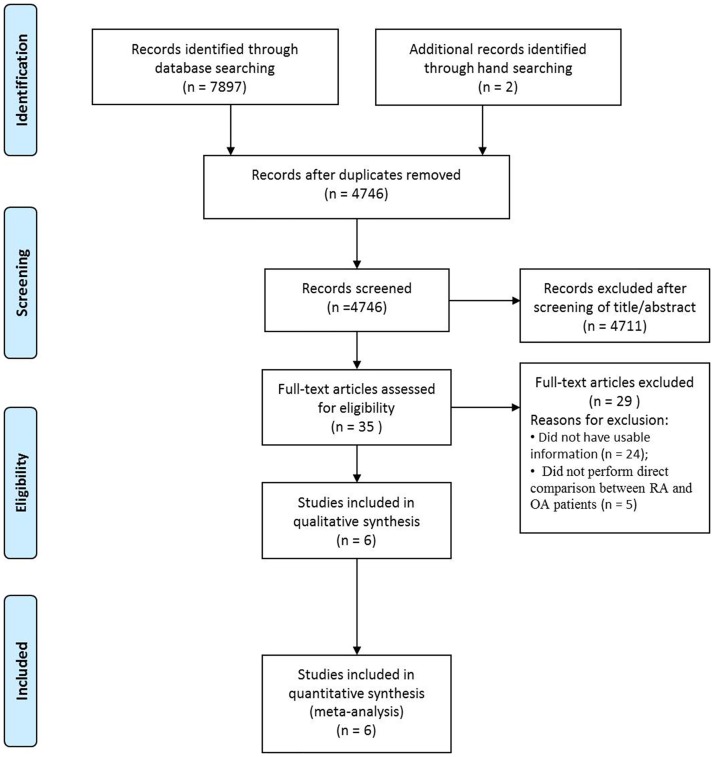
PRISMA (Preferred Reporting Items for Systematic reviews and Meta-analyses) flow diagram of the identification and selection of the studies included in this meta-analysis.

Fig 1 shows the details of study identification, inclusion, and exclusion. Electronic searches yielded 2291 studies in PubMed (MEDLINE), 1951 in EMBASE, 328 in Web of Science, 3115 in SCOPUS, and 212 in the Cochrane Library. Two additional publications were identified through manual searching. After 3153 duplicates were removed, 4746 studies remained. Of these, 4711 were excluded as it was clear from their abstract and title that they did not fulfil the selection criteria. An additional 29 studies were excluded based on unusable information and inappropriate between-group comparisons (S1 Excluded studies). Finally, six studies [7–12] were included in this meta-analysis. Dataset used in this study is given in S1 Raw data.

### Characteristics and Quality of the included studies

Of the six included studies, two compared all four parameters, including asymptomatic and symptomatic DVTs and VTEs, in RA and OA groups; three compared two parameters, symptomatic DVTs and VTEs; and one compared symptomatic VTEs alone (Table 1).

**Table 1:** Characteristics of the studies included in the meta-analysis.

Study	Year	Study type	Sample size		Quality score	Diagnostic tool	Measured parameters
			RA	OA			
LoVerde et al. [10]	2015	RCT	159	318	6	Not stated	SDVT, SVTE
Izumi et al. [7]	2015	RCT	204	1084	6	US	ADVT, SDVT, AVTE, SVTE
Niki et al. [8]	2010	RCS	238	169	8	US	ADVT, SDVT, AVTE, SVTE
Ravi et al. [9]	2014	PCS	2692	59564	8	Not stated	SVTE
Schnaser et al. [11]	2015	RCT	209916	6616985	4	Not stated	SDVT, SVTE
Stundner et al. [12]	2014	RCT	42	47	4	Venography	SDVT, SVTE

Abbreviations: RCT, randomized controlled trial; RCS, retrospective comparative study; PCS, prospective comparative study; US, ultrasonography; ADVT, asymptomatic deep vein thrombosis; SDVT, symptomatic deep vein thrombosis; AVTE, asymptomatic venous thromboembolism; SVTE, symptomatic venous thromboembolism.

All six studies compared infection and revision rates in RA and OA groups, as well as providing detailed demographic data of these patients. None assessed possible confounding factors. Of the six studies, four were assessed as being of high quality (Newcastle-Ottawa Scale ≥5, Table 1)

### Deep vein thrombosis

**Fig 2:**
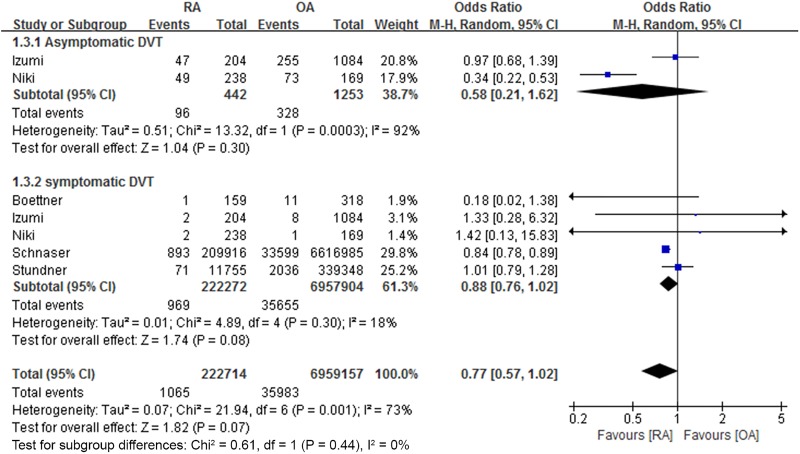
Forest plot showing differences in the rate of deep vein thrombosis after total knee arthroplasty between patients with rheumatoid arthritis (RA) and osteoarthritis (OA).

Of the six studies, two compared rates of asymptomatic and symptomatic DVT, and four compared rates of symptomatic DVT after primary TKA in patients with RA and OA. The postoperative rates of asymptomatic (96/442 [21.7%] vs. 328/1253 [26.2%]; OR 0.58, 95% CI: 0.21 to 1.62; P = 0.30) and symptomatic (969/222,272 [0.4%] vs. 35,655/6,957,904 [0.5%]; OR 0.88, 95% CI: 0.76 to 1.02; P = 0.08) DVT were similar in the RA and OA groups, as were the combined rates of asymptomatic and symptomatic DVT (1065/222,714 [0.5%] vs. 35,983/6,959,157 [0.5%]; OR 0.77, 95% CI: 0.57 to 1.02; P = 0.07, Fig 2).

### Venous thromboembolism

**Fig 3:**
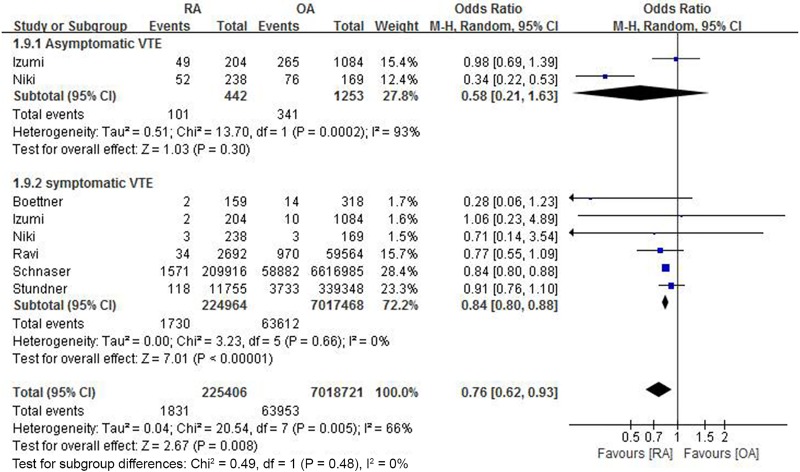
Forest plot showing differences in the rate of venous thromboembolism after total knee arthroplasty between patients with rheumatoid arthritis (RA) and osteoarthritis (OA).

Although the rates of asymptomatic VTE (DVT or PE) after TKA were similar in the RA and OA groups (101/442 [22.9%] vs. 341/1253 [27.2%]; OR 0.58, 95% CI: 0.21 to 1.63; P = 0.30), the rate of symptomatic VTE was significantly lower in patients with RA than in those with OA (1730/224,964 [0.8%] vs. 63,612/7,017,468 [0.9%]; OR 0.84, 95% CI: 0.80 to 0.88; P<0.001). Pooled data of these two subgroup analyses showed that the combined rate of asymptomatic and symptomatic VTE was significantly lower in the RA than in the OA group (1831/225,406 [0.8%] vs. 63,953/7,018,721 [0.9%]; OR 0.76, 95% CI: 0.62 to 0.93; P = 0.008, Fig 3).

### Meta-regression analyses

Table 2 shows the results of the meta-regression analysis. Age, gender, and effect size showed no significant associations with differences in rates of post-TKA DVT and VTE in the RA and OA groups. This finding indicated that the results of the current meta-analysis were not biased by the demographic characteristics of the participants in the included studies.

**Table 2:** Meta-regression analysis comparing associations of age and gender with rates of deep vein thrombosis (DVT) and venous thromboembolism (VTE) after total knee arthroplasty in patients with rheumatoid arthritis and osteoarthritis.

Variable	Coefficient	Standard error	P value	95% confidence interval
Difference in DVT incidence after TKA in RA and OA patients				
Age	−0.016	0.016	0.921	−0.045 to 0.041
Gender	0.001	0.012	0.911	−0.031 to 0.034
Difference in VTE incidence after TKA in RA and OA patients				
Age	−0.002	0.020	0.933	−0.064 to 0.061
Gender	0.001	0.138	0.921	−0.042 to 0.045

## Discussion

The most important findings of this meta-analysis were that the incidence of DVT after primary TKA did not differ between groups of patients with RA and OA, whereas the incidence of VTE after primary TKA was lower in the RA than in the OA group. The latter finding therefore contradicts our hypothesis, that the incidence of post-TKA thrombotic complications would be higher in patients with RA than in patients with OA.

Although it is unclear if RA is a risk factor for the development of VTE after TKA, the chronic inflammation associated with RA suggests that thrombi may form more readily in these patients than in patients with OA. Reduced mobility due to RA may lead to venous stasis.[13] In addition, inflammation may cause endothelial damage to vessel walls, leading to endothelial dysfunction in both arteries and veins.[14] Chronic inflammation may also alter thrombotic responses by upregulating precoagulants,[15] downregulating anticoagulants and suppressing fibrinolysis,[16] enhancing blood coagulability. The combination of venous stasis, vascular endothelial dysfunction due to damage to vessel walls, and blood hypercoagulability in patients with RA may favor the development of VTE.[5] Several studies also confirmed that the risks of DVT and PE significantly increased by 1.9–3.36 and 2.07–2.25 fold, respectively, in patients with RA compared with those of the general population.[17,18]

Despite these apparent links between RA and VTE, previous studies comparing the incidence of post-TKA DVT and VTE in RA and OA patients have yielded contradictory results. Our meta-analysis showed no differences in the rates of DVT and asymptomatic VTE after TKA between RA and OA patients, and demonstrated that the incidence of symptomatic VTE was lower in RA than in OA patients. These findings, which were contrary to our expectations, may have resulted from confounding factors. Because of their underlying disease, patients with RA are more likely than patients with OA to take medications such as methotrexate, sulfasalazine, and nonsteroidal anti-inflammatory drugs (NSAIDs).[19] These medications may offset the VTE-prone environment of RA, with NSAIDs especially having antiplatelet activity.[8] We could also not entirely rule out selection bias, especially in age. At the time of TKA, patients with RA tend to be younger than those with OA [9,20], which may reduce the relative risk of VTE in patients with RA. Conversely, the higher incidence of VTE in RA than in OA patients after TKA in several previous studies may have been due to differences in sex distribution of these two diseases, with the proportion of females being higher in RA than in OA.[7,20] The meta-regression analysis performed in this study to evaluate the effect of age distribution and gender ratio on VTE incidence after TKA showed that age and gender in the RA and OA cohorts did not correlate significantly with rates of DVT and VTE.

Caution should be exercised in interpreting comparative rates of VTE in previous studies, because several of these studies focused only on symptomatic DVT and/or PE. Asymptomatic distal DVT is generally considered self-limiting, stable and associated with a lower risk of embolic complications than proximal DVT.[21] However, the likelihood of proximal extension of distal DVT was reported to be as high as 20%[22] when distal DVT was left untreated.[23] Proximal extension is likely to cause serious embolic complications, including PE. In addition, we could not determine whether all asymptomatic DVTs in the studies included in this meta-analysis were distal, generally regarded as below the popliteal area. Because asymptomatic silent PE can lead to serious complications, including symptomatic recurrent PE and pulmonary hypertension, it has been recommended that, similar to symptomatic PE, asymptomatic silent PE should be treated with anticoagulants.[23] These findings suggest that the incidence of asymptomatic VTE (DVT and PE) should be evaluated when comparing the rates of actual VTE after TKA in RA and OA patients. The strength of this meta-analysis was that it included all studies that reported rates of both asymptomatic and symptomatic VTE, and that it included subgroup analyses comparing rates of asymptomatic and symptomatic VTE in these patient groups.

This study had several limitations. A key limitation was the potential risk of bias caused by possible unmeasured or unknown confounders, including smoking status, body mass index, medication data, and physical activity levels of patients in each included study. In RA patients, disease severity was not consistent among the included studies. In addition, the method of diagnosing DVT was not consistent across studies. Although venography is generally accepted as the gold standard in detecting DVT,[7] ultrasonography is widely used and has been shown to be accurate in the postoperative diagnosis of DVT, even in asymptomatic orthopedic patients.[24,25] These factors may explain, at least in part, some of the heterogeneities in the results of this meta-analysis. Finally, the findings of this meta-analysis may have been heavily influenced by the results of the study with the largest sample size, which were assigned the greatest weight.

## Conclusions

The incidence of DVT after primary TKA in RA patients was similar to that in OA patients. In contrast, the incidence of VTE (DVT plus PE) after primary TKA was lower in RA than in OA patients, despite chronic inflammation favoring thrombus formation in patients with RA.

## Supporting Information

S1 PRISMA Checklist (PDF)

S1 Search strategy (DOC)

S1 Excluded studies (DOC)

S1 Raw data (XLS)
