# Supplementary material for: Incidence of Deep Vein Thrombosis and Venous Thromboembolism following TKA in Rheumatoid Arthritis versus Osteoarthritis: A Meta-Analysis
Source: PLoS One. 2016 Dec 2;11(12):e0166844. doi: 10.1371/journal.pone.0166844 (PMC5135053; doi:10.1371/journal.pone.0166844)
Supplement: S1 Search strategy — (DOC) [file pone.0166844.s002.doc]

MEDLINE

1. ((Rheumatoid[tiab] OR Degenerative[tiab]) AND (Arthritis[tiab] OR Arthritides[tiab])) OR (Osteoarthritides[tiab] OR Osteoarthrosis[tiab] OR Osteoarthroses[tiab]) 97485
2. ("Arthritis, Rheumatoid"[Mesh]) OR "Osteoarthritis"[Mesh] 141054
3. 1 OR 2 170710
4. "Arthroplasty, Replacement, Knee"[Mesh]) OR "Knee Prosthesis"[Mesh] 20881
5. (knee[tiab] OR knees[tiab]) AND (Arthroplasty[tiab] OR Arthroplasties[tiab] OR Replacement[tiab] OR Replacements[tiab] OR Prostheses[tiab] OR Prosthesis[tiab]) 27216
6. 4 OR 5 30825
7. 3 AND 6 7419
8. "Postoperative Complications"[Mesh:NoExp] OR "adverse effects"[Subheading] 2070613
9. Complication[tiab] OR Complications[tiab] OR "adverse effects"[tiab] 856057
10. (("Pulmonary Embolism"[Mesh:NoExp]) OR "Venous Thromboembolism"[Mesh]) OR "Venous Thrombosis"[Mesh] 79224
11. (Deep-Venous[tiab] OR Venous[tiab] OR Pulmonary[tiab] OR vein[tiab] OR peripheral[tiab] OR Deep-Vein[tiab]) AND (Thrombosis[tiab] OR Thromboses[tiab] OR Thrombus[tiab] OR Thromboembolism[tiab] OR Embolisms[tiab] OR Embolisms[tiab] OR Embolus[tiab] OR Thromboembolisms[tiab]) 78285
12. 8 OR 9 OR 10 OR 11 2709929
13. 7 AND 12 2626
14. knee[tiab] OR knees[tiab] 111291
15. 13 AND 14 2519
16. 15 NOT ("review"[Publication Type] OR "review literature as topic"[MeSH Terms]) 2291

EMBASE

1. ((Rheumatoid:ab,ti OR Degenerative:ab,ti) AND (Arthritis:ab,ti OR Arthritides:ab,ti)) OR (Osteoarthritides:ab,ti OR Osteoarthrosis:ab,ti OR Osteoarthroses:ab,ti) 128776
2. 'rheumatoid arthritis'/exp OR 'osteoarthritis'/exp 254138
3. 1 OR 2 270257
4. (knee:ab,ti OR knees:ab,ti) AND (Arthroplasty:ab,ti OR Arthroplasties:ab,ti OR Replacement:ab,ti OR Replacements:ab,ti OR Prostheses:ab,ti OR Prosthesis:ab,ti) 33203
5. 'knee arthroplasty'/exp OR 'knee prosthesis'/exp 34212
6. 4 OR 5 41687
7. 3 AND 6 11228
8. knee:ab,ti OR knees:ab,ti 138159
9. Complication:ab,ti OR Complications:ab,ti OR "adverse effects":ab,ti 1074295
10. 'postoperative complication'/de OR 'postoperative thrombosis'/exp OR 'lung embolism'/exp OR 'venous thromboembolism'/exp OR 'embolism'/de OR 'vein embolism'/exp OR 'vein thrombosis'/de OR 'deep vein thrombosis'/exp OR 'lower extremity deep vein thrombosis'/exp OR 'upper extremity deep vein thrombosis'/exp 43332
11. (Deep-Venous:ab,ti OR Venous:ab,ti OR Pulmonary:ab,ti OR vein:ab,ti OR peripheral:ab,ti OR Deep-Vein:ab,ti) AND (Thrombosis:ab,ti OR Thromboses:ab,ti OR Thrombus:ab,ti OR Thromboembolism:ab,ti OR Embolisms:ab,ti OR Embolisms:ab,ti OR Embolus:ab,ti OR Thromboembolisms:ab,ti)109742
12. 9-11/OR 1380476
13. 7 AND 12 2295
14. 13 AND 8 2130
15. 14 NOT 'review'/it 1951

COCHRANE

1. ((Rheumatoid or Degenerative) and (Arthritis or Arthritides)) or (Osteoarthritides or Osteoarthrosis or Osteoarthroses):ti,ab,kw (Word variations have been searched) 8340

2. MeSH descriptor: [Arthritis, Rheumatoid] explode all trees 4467

3. MeSH descriptor: [Osteoarthritis] explode all trees 4134

4. 2 OR 3 8292

5. 1 OR 4 12120

6. MeSH descriptor: [Arthroplasty, Replacement, Knee] explode all trees 2058

7. MeSH descriptor: [Knee Prosthesis] explode all trees 670

8. 6 OR 7 2360

9. (knee or knees) and (Arthroplasty or Arthroplasties or Replacement or Replacements or Prostheses or Prosthesis):ti,ab,kw (Word variations have been searched) 4338

10. 8 OR 9 4338

11. 5 AND 10 738

12. MeSH descriptor: [Postoperative Complications] this term only 15770

13. Complication or Complications or "adverse effects":ti,ab,kw (Word variations have been searched) 94488

14. MeSH descriptor: [Pulmonary Embolism] this term only 982

15. MeSH descriptor: [Venous Thromboembolism] explode all trees 514

16. MeSH descriptor: [Venous Thrombosis] explode all trees 2448

17. 14 OR 15 OR 16 3358

18. (Deep-Venous or Venous or Pulmonary or vein or peripheral or Deep-Vein) and (Thrombosis or Thromboses or Thrombus or Thromboembolism or Embolisms or Embolisms or Embolus or Thromboembolisms):ti,ab,kw (Word variations have been searched) 8435

19. 12 OR 13 OR 17 OR 18 100383

20. 11 AND 19 228

21. knee or knees:ti,ab,kw (Word variations have been searched) 14085

22. 20 AND 21 229

23. 22/TRIALS 212

WOS

1. TOPIC: (((Rheumatoid OR Degenerative ) AND (Arthritis OR Arthritides )) OR (Osteoarthritides OR Osteoarthrosis OR Osteoarthroses )) OR TITLE: (((Rheumatoid OR Degenerative ) AND (Arthritis OR Arthritides )) OR (Osteoarthritides OR Osteoarthrosis OR Osteoarthroses )) 143329

2. TOPIC: ((knee OR knees ) AND (Arthroplasty OR Arthroplasties OR Replacement OR Replacements OR Prostheses OR Prosthesis )) OR TITLE: ((knee OR knees ) AND (Arthroplasty OR Arthroplasties OR Replacement OR Replacements OR Prostheses OR Prosthesis )) 31785

3. 1 AND 2 1762

4. TOPIC: (Complication OR Complications OR "adverse effects") OR TITLE: (Complication OR Complications OR "adverse effects") 653159

5. TOPIC: ((Deep-Venous OR Venous OR Pulmonary OR vein OR peripheral OR Deep-Vein) AND (Thrombosis OR Thromboses OR Thrombus OR Thromboembolism OR Embolisms OR Embolisms OR Embolus OR Thromboembolisms)) OR TITLE: ((Deep-Venous OR Venous OR Pulmonary OR vein OR peripheral OR Deep-Vein) AND (Thrombosis OR Thromboses OR Thrombus OR Thromboembolism OR Embolisms OR Embolisms OR Embolus OR Thromboembolisms)) 103037

6. 4 OR 5 734491

7. 3 AND 6 368

8. knee OR knees 117448

9. 7 AND 8 368

10. 9 [excluding] DOCUMENT TYPES: ( REVIEW ) 328

SCOPUS

1. TITLE-ABS-KEY ( ( ( rheumatoid OR degenerative ) AND ( arthritis OR arthritides ) ) OR ( osteoarthritides OR osteoarthrosis OR osteoarthroses ) ) 175239

2. INDEXTERMS(("Arthritis, Rheumatoid") OR "Osteoarthritis") 155479

3. 1 OR 2 234227

4. INDEXTERMS(("Arthroplasty, Replacement, Knee") OR "Knee Prosthesis") 22712

5. TITLE-ABS-KEY((knee OR knees) AND (Arthroplasty OR Arthroplasties OR Replacement OR Replacements OR Prostheses OR Prosthesis)) 47088

6. 4 OR 5 47088

7. 3 AND 6 11845

8. INDEXTERMS("Postoperative Complications") 419415

9. TITLE-ABS-KEY(Complication OR Complications OR "adverse effects") 1622502

10. INDEXTERMS((("Pulmonary Embolism") OR "Venous Thromboembolism") OR "Venous Thrombosis" ) 63694

11. TITLE-ABS-KEY((Deep-Venous OR Venous OR Pulmonary OR vein OR peripheral OR Deep-Vein ) AND (Thrombosis OR Thromboses OR Thrombus OR Thromboembolism OR Embolisms OR Embolisms OR Embolus OR Thromboembolisms )) 211545

12. 10 OR 11 OR 8 OR 9 1771521

13. 7 AND 12 3442

14. TITLE-ABS-KEY(knee OR knees) 183345

15. 13 AND 14 3442

16. 15 AND ( EXCLUDE ( DOCTYPE , "re" ) ) 3115

MEDLINE 2291

EMBASE 1951

COCHRANE 212

WOS 328

SCOPUS 3115
