# Supplementary material for: Incidence of Deep Vein Thrombosis and Venous Thromboembolism following TKA in Rheumatoid Arthritis versus Osteoarthritis: A Meta-Analysis
Source: PLoS One. 2016 Dec 2;11(12):e0166844. doi: 10.1371/journal.pone.0166844 (PMC5135053; doi:10.1371/journal.pone.0166844)
Supplement: S1 Excluded studies — (DOC) [file pone.0166844.s003.doc]

Excluded studies

1. Buchheit J, Serre A, Bouilloux X, Puyraveau M, Jeunet L, Garbuio P. Cementless total knee arthroplasty in chronic inflammatory rheumatism. *Eur J Orthop Surg Traumatol.* 2014;24(8):1489-1498. No direct comparison between RA and OA

2. Centeno CJ, Hanson Jr RW, Schultz JR, Newton BJ, Reischling P. Treatment of knee osteoarthrosis with autologous marrow concentrate. *Clinical Journal of Sport Medicine.* 2014;24(2):181-182. No usable information

3. Chen JY, Chin PL, Moo IH, et al. Intravenous versus intra-articular tranexamic acid in total knee arthroplasty: A double-blinded randomised controlled noninferiority trial. *Knee.* 2016;23(1):152-156. No direct comparison between RA and OA

4. Chowdhry M, Bamne AB, Na YG, Kang YG, Kim TK. Prevalence and predictors of post-operative coronal alignment outliers and their association with the functional outcomes in navigated total knee arthroplasty. *J Arthroplasty.* 2014;29(12):2357-2362. No usable information

5. Chung LH, Chen WM, Chen CF, Chen TH, Liu CL. Deep vein thrombosis after total knee arthroplasty in asian patients without prophylactic anticoagulation. *Orthopedics.* 2011;34(1):15. No usable information

6. da Cunha BM, de Oliveira SB, Santos-Neto L. Incidence of infectious complications in hip and knee arthroplasties in rheumatoid arthritis and osteoarthritis patients. *Rev Bras Reumatol.* 2011;51(6):609-615. No direct comparison between RA and OA

7. Davies R, Galloway J, Watson KD, Lunt M, Symmons D, Hyrich KL. Are venous thrombotic events increased in patients with rheumatoid arthritis treated with anti-tnf therapy? Results from the British society for rheumatology biologics register (BSRBR). *Rheumatology.* 2011;50:iii123-iii124. No direct comparison between RA and OA

8. Hitzl W, Sattler M, Eckstein F, Cotofana S. The projected numbers of total knee replacement in Austria-from 2010 to 2075. *Osteoarthritis and Cartilage.* 2014;22:S216-S217. No usable information

9. Johnson B, Goodman SM, Alexiades M, Mandl LA. Perioperative use of anti-tnf medications in patients with rheumatoid arthritis undergoing total knee replacement. *Arthritis and Rheumatism.* 2011;63(10). No usable information

10. Kaplunov OA, Mikhin IV, Biriukov SN. [Blood loss during total knee-joint replacement: a combination of anticoagulant and hemostatic techniques]. *Khirurgiia (Mosk).* 2014(12):41-45. No usable information

11. Kotela A, Kotela I. Patient-specific computed tomography based instrumentation in total knee arthroplasty: a prospective randomized controlled study. *Int Orthop.* 2014;38(10):2099-2107. No usable information

12. Mameli A, Marongiu F. Thromboembolic disease in patients with rheumatoid arthritis undergoing joint arthroplasty: Update on prophylaxes. *World Journal of Orthopaedics.* 2014;5(5):645-652. No usable information

13. Michaud K, Fehringer EV, Garvin K, O'Dell JR, Mikuls TR. Rheumatoid arthritis patients are not at increased risk for 30-day cardiovascular events, infections or mortality following total joint arthroplasty. *Arthritis Research & Therapy.* 2013:R195. No usable information

14. Momohara S, Kawakami K, Iwamoto T, et al. Prosthetic joint infection after total hip or knee arthroplasty in rheumatoid arthritis patients treated with nonbiologic and biologic disease-modifying antirheumatic drugs. *Modern Rheumatology.* 2011;21(5):469-475.

No usable information

15. Nagase Y, Yasunaga H, Horiguchi H, et al. Risk factors for pulmonary embolism and the effects of fondaparinux after total hip and knee arthroplasty: a retrospective observational study with use of a national database in Japan. *J Bone Joint Surg Am.* 2011;93(24):e146.

No usable information

16. Naili JE, Iversen MD, Esbjörnsson AC, et al. Challenging the effect of hip and knee replacement for osteoarthritis-a prospective one year follow-up study of 64 patients evaluating function and gait dynamics. *Osteoarthritis and Cartilage.* 2016;24:S111. No usable information

17. Niinimäki T, Eskelinen A, Mäkelä K, Ohtonen P, Puhto AP, Remes V. Unicompartmental knee arthroplasty survivorship is lower than TKA survivorship: A 27-year finnish registry study. *Clinical Orthopaedics and Related Research.* 2014;472(5):1496-1501. No usable information

18. Oremus K, Sostaric S, Trkulja V, Haspl M. Influence of tranexamic acid on postoperative autologous blood retransfusion in primary total hip and knee arthroplasty: A randomized controlled trial. *Transfusion.* Vol 54; 2014:31-41. No usable information

19. Park IH, Lee SC, Park IS, et al. Asymptomatic peripheral vascular disease in total knee arthroplasty: preoperative prevalence and risk factors. *J Orthop Traumatol.* 2015;16(1):23-26.

20. Pedersen AB, Mehnert F, Johnsen SP, Husted S, Sorensen HT. Venous thromboembolism in patients having knee replacement and receiving thromboprophylaxis: a Danish population-based follow-up study. *J Bone Joint Surg Am.* 2011;93(14):1281-1287. No usable information

21. Pethes T, Bejek Z, Kiss RM. The effect of knee arthroplasty on balancing ability in response to sudden unidirectional perturbation in the early postoperative period. *Journal of Electromyography and Kinesiology.* 2015;25(3):508-514. No usable information

22. Phillips JE, Crane TP, Noy M, Elliott TSJ, Grimer RJ. The incidence of deep prosthetic infections in a specialist orthopaedic hospital. A 15-year prospective survey. *Journal of Bone and Joint Surgery - Series B.* 2006;88(7):943-948. No usable information

23. Polkowski GG, Duncan ST, Bloemke AD, Schoenecker PL, Clohisy JC. Screening for Deep Vein Thrombosis After Periacetabular Osteotomy in Adult Patients: Is It Necessary? *Clinical Orthopaedics and Related Research.* 2014;472(8):2500-2505. No usable information

24. Pritchett JW. Bicruciate-retaining Total Knee Replacement Provides Satisfactory Function and Implant Survivorship at 23 Years. *Clinical Orthopaedics and Related Research.* 2015;473(7):2327-2333. No usable information

25. Song K, Rong Z, Yang X, et al. Early Pulmonary Complications following Total Knee Arthroplasty under General Anesthesia: A Prospective Cohort Study Using CT Scan. *Biomed Res Int.* 2016;2016:4062043. No usable information

26. Song K, Xu Z, Rong Z, et al. The incidence of venous thromboembolism following total knee arthroplasty: A prospective study by using computed tomographic pulmonary angiography in combination with bilateral lower limb venography. *Blood Coagulation and Fibrinolysis.* 2016;27(3):266-269. No usable information

27. Treuter S, Schuh A, Hönle W, Ismail MS, Chirag TN, Fujak A. Long-term results of total knee arthroplasty following high tibial osteotomy according to Wagner. *International Orthopaedics.* 2012;36(4):761-764. No usable information

28. Waldenberger P, Chemelli A, Hennerbichler A, et al. Transarterial embolization for the management of hemarthrosis of the knee. *European Journal of Radiology.* 2012;81(10):2737-2740. No usable information

29. Yan DL, Yang J, Pei FX. Total knee arthroplasty treatment of rheumatoid arthritis with severe versus moderate flexion contracture. *Journal of Orthopaedic Surgery and Research.* 2013;8.

No direct comparison between RA and OA
